# Protective but Costly: The Impact of Behavioral Immune System Reactivity on Mental Health

**DOI:** 10.3390/ijerph22060900

**Published:** 2025-06-05

**Authors:** Ivana Hromatko, Una Mikac, Anita Lauri Korajlija, Nataša Jokić-Begić, Tanja Jurin, Meri Tadinac

**Affiliations:** Department of Psychology, Faculty of Humanities and Social Sciences, University of Zagreb, 10000 Zagreb, Croatia; umikac@ffzg.hr (U.M.); alauri@ffzg.hr (A.L.K.); njbegic@ffzg.hr (N.J.-B.); tjurin@ffzg.hr (T.J.); mtadinac@ffzg.unizg.hr (M.T.)

**Keywords:** behavioral immune system, anxiety, stress, depression, OC symptoms, COVID-19 pandemic, costly adaptations

## Abstract

The behavioral immune system (BIS) refers to a set of evolved psychological mechanisms designed to detect cues of potential pathogen threat and trigger self-protective, avoidant behaviors. However, like all adaptations, the BIS carries potential costs alongside its benefits. This study aimed to examine the impact of BIS-related processes on mental health outcomes—including depression, anxiety, stress, and obsessive–compulsive symptomatology—during the COVID-19 pandemic. Data were collected online at two time points: the onset of the pandemic (May 2020; *n* = 990; 86% women) and at the end of its first year (November/December 2020; a subsample of the original participants, *n* = 182). Hierarchical regression analyses were conducted, entering socioeconomic variables and general quality of life in the first block, followed by BIS-related variables (germ aversion and perceived infectability) in the second block. Results showed that socioeconomic status and general quality of life were significant predictors of mental health difficulties at both time points, consistent with prior findings on the harmful effects of environmental and social stressors. Importantly, BIS variables also emerged as significant and independent predictors of mental health outcomes—including the development of obsessive–compulsive symptoms—highlighting the potential psychological costs of pathogen-avoidant motivations governed by the BIS.

## 1. Introduction

### 1.1. The Behavioral Immune System: Mechanisms and Mental Health Implications

Throughout human evolutionary history, various parasitic organisms exerted substantial selective pressures, resulting in the development of complex and highly efficient physiological defense mechanisms—collectively known as the immune system. While effective, these immunological responses are metabolically costly, can temporarily impair functioning (e.g., through fever or cytokine-induced sickness behavior), and may even cause long-term harm in cases of autoimmune reactions [1,2]. These costs can compete with other vital functions, such as reproduction and somatic maintenance, thereby generating trade-offs in resource allocation. For instance, organisms may face a choice between investing in immune defense or in life history traits like reproduction, potentially reducing reproductive success as energy is redirected. Natural selection, therefore, likely favored individuals capable of optimizing this balance based on environmental cues such as pathogen prevalence and resource availability. In high-parasite environments, stronger immune responses might be favored, even at the cost of reproductive output. Conversely, in low-parasite environments, prioritizing reproductive investment over costly immune activity may prove advantageous.

Unlike the physiological immune system, which reacts only once pathogens have entered the body, the behavioral immune system (BIS) proactively identifies cues of potential contamination—such as contagious individuals or objects—and elicits prophylactic, avoidant behaviors [3,4,5]. Research indicates that humans can detect subtle visual, auditory, and olfactory indicators of illness in others [6,7,8]. Upon perceptual detection, emotional and cognitive processes are triggered to motivate pathogen avoidance [5]. Remarkably, merely viewing images of pathogens can elevate body temperature and increase salivary levels of TNF-alpha, albumin [9], and IL-6 [10], suggesting that perceived pathogen threats may initiate not only behavioral but also physiological immune responses.

Cues related to disease have high attentional salience and are easily retained in memory [11,12,13]. Since BIS activation is primarily emotionally driven, it operates largely outside of conscious awareness and automatically influences various domains of social cognition—including ethnocentrism, conformism, prejudice, political orientation, and mate preferences [14,15,16,17,18,19,20,21,22,23]. However, the mental health implications of these automatic responses, particularly when they become dysregulated, have received limited attention. Drawing an analogy with the well-known costs of physiological immune function, heightened BIS reactivity (i.e., perceiving excessive disease threats) may also come with trade-offs—such as increased social withdrawal, which could hinder social bonding and mating opportunities.

The BIS is flexible and context-dependent. Individuals who perceive themselves as more susceptible to disease are more likely to be sensitive to pathogen cues in their environment [24]. While epidemics are not new in evolutionary history, the COVID-19 pandemic was unprecedented in its global scale and prolonged duration. Prior to COVID-19, BIS activation was usually studied via priming in laboratory settings. The pandemic, however, offered a unique opportunity to observe BIS processes in a real-world context. Findings indicate that BIS-related variables predicted numerous pandemic-related behaviors, such as compliance with public health measures, vaccine attitudes, illness appraisal, and changes in social cognition [25,26,27,28,29,30,31]. Furthermore, scores on measures like disgust sensitivity, germ aversion, and perceived infectability significantly increased compared to pre-pandemic levels [25,32,33], reflecting the BIS’s contextually responsive nature [34]. These reactions were largely driven by information signaling immediate risk, even in the absence of direct sensory cues of infection.

Although BIS activation is presumably less metabolically costly than physiological immune responses, the principle of “no free lunch” applies to both biological and psychological adaptations. Darwinian psychiatry has identified several potential costs of psychological defenses, such as the development of phobias and obsessive–compulsive symptoms [35]. Chronic BIS activation is associated with elevated stress and anxiety—states known to increase the risk of mental illness and mortality through mechanisms such as allostatic load and chronic inflammation [36,37,38,39,40,41,42,43,44,45,46,47]. Avoidance behaviors triggered by BIS activation—such as steering clear of potentially contaminated environments or individuals—may also lead to reduced participation in activities beneficial to mental and physical health, including social interaction and exercise. Individuals with hyperactive BIS responses may become hypersensitive to contamination cues, leading to heightened anxiety and the adoption of compulsive behaviors like excessive handwashing or checking. Prior research has shown that disgust propensity and germ aversion predict contamination-based obsessions and compulsions in both clinical and non-clinical populations [48,49]. Such behaviors temporarily reduce anxiety, reinforcing the compulsions over time. Thus, excessive BIS reactivity may contribute to the development or exacerbation of obsessive–compulsive symptomatology.

### 1.2. Socioeconomic Status, Behavioral Immune System, and Mental Health

Socioeconomic status (SES) is a well-established determinant of both physical and mental health, with numerous studies confirming its pervasive and long-lasting effects [39,40,41,42,50,51,52,53,54,55]. Lower SES is consistently linked to poorer overall health, higher morbidity rates, and shorter life expectancy. Mechanisms through which SES influences health include limited access to medical care, increased exposure to environmental hazards, and engagement in less health-promoting behaviors. Individuals of lower SES frequently face heightened financial stress, substandard living conditions, and restricted access to nutritious food and preventive care—all of which contribute to a higher risk of developing chronic diseases such as cardiovascular disorders, diabetes, and respiratory illness.

Beyond physical health, SES also plays a critical role in psychological well-being [45,46,47,56,57]. Chronic stress associated with poverty, unemployment, and social marginalization has been linked to increased anxiety, depression, and psychological distress. According to the allostatic load model, prolonged socioeconomic adversity disrupts physiological systems (e.g., the HPA axis), ultimately impairing both physical and mental health [36,37,38,58,59]. These conditions may also heighten vigilance toward environmental threats, potentially sensitizing individuals to pathogen cues and thus intensifying BIS activity. This interplay between low SES, chronic stress, and BIS reactivity is especially relevant in the context of the COVID-19 pandemic, which introduced widespread environmental and social disruptions—affecting health, the economy, education, and more. Given these converging stressors, an increase in mental health issues during the pandemic was, arguably, inevitable.

Accordingly, the present study aimed to examine how BIS reactivity, socioeconomic status, and overall quality of life contributed to mental health outcomes during the COVID-19 pandemic. The BIS was operationalized, according to Duncan, Schaller, and Park [60], using two core constructs: germ aversion and perceived infectability. Germ aversion captures affective and behavioral discomfort in situations perceived to involve pathogen exposure (e.g., unease at sharing a glass), reflecting motivational tendencies to avoid contamination cues. Perceived infectability assesses individuals’ beliefs about their susceptibility to infectious disease, based on personal health history or constitution (e.g., “In general, I am very susceptible to colds, flu, and other infectious diseases”). These dimensions represent complementary facets of BIS functioning—one focused on situational reactivity, the other on dispositional perceptions of vulnerability. Both have been psychometrically validated and are associated with pathogen-avoidant behaviors and attitudes.

The study was conducted online in Croatia at two time points: the beginning of the COVID-19 pandemic (May 2020) and the end of its first year (November–December 2020). This longitudinal approach allowed us to assess both immediate and longer-term effects of BIS activation. We hypothesized that lower SES and poorer general quality of life would predict worse mental health outcomes and that BIS-related variables would independently explain additional variance in mental health indicators beyond that accounted for by SES and quality of life.

## 2. Materials and Methods

This study was part of a larger research project titled “How Are We? Life in Croatia in the Age of Coronavirus”. Data were collected through an online survey administered at two time points (May 2020 and November/December 2020), with participation from over 4000 individuals across various population subgroups. The survey link was distributed via various social media platforms as well as through mainstream media outlets, online news portals, and similar channels.

The survey began with a general section that all participants completed, which included demographic questions and measures of stress, mental health, and overall quality of life. Following this, participants were invited to continue with additional sections tailored to specific topics (e.g., work experiences during the pandemic, partner relationships, changes in daily routines) or targeted at particular subgroups (e.g., students, parents, seniors, couples, workers). Some sections were universally available, while others were selectively presented based on participants’ demographic responses.

Participants had the option to complete one, several, or none of the additional modules, depending on their relevance and interest. For instance, a working parent might choose to complete sections on general mental health, work-related stress, parenting during lockdown, and partner relationships. At the end of the survey, participants were given the option to provide their email address if they wished to be contacted for follow-up participation at the second time point.

The project received ethical approval from the Ethics Committee of the Faculty of Humanities and Social Sciences, University of Zagreb, and was conducted in accordance with established ethical guidelines for research involving human participants.

### 2.1. Participants

Participants were recruited using a convenience sampling method, primarily through snowball sampling and online dissemination. No exclusion criteria were applied, allowing for a diverse and self-selected sample. The present study is based on responses from participants who chose to complete the sections on general mental health and behavioral immune system measures at the first time point (*n* = 990). For the second time point, only data from returning participants—those who took part in both waves of data collection—are included in the analyses presented here (*n* = 182). All participants were living in Croatia at the time of data collection. The majority were women (over 85%) with a mean age of around 35–36 years. Most participants had at least a high school education, with graduate degrees being the most common. Relationship status was fairly evenly distributed among single, cohabiting, and non-cohabiting individuals. The detailed demographic composition of the sample (and its subsample at the second time point) is presented in Table 1.

### 2.2. Instruments

Sociodemographic information: Participants provided information on their gender, age, education level, relationship status, parental status, financial status, and general quality of life. General quality of life was assessed using a single-item measure: “Overall, how satisfied are you with your general quality of life?”, with responses ranging from 0 (“not at all”) to 10 (“completely satisfied”).

Perceived Vulnerability to Disease Scale (PVD) [60]: This scale comprises two subscales: (1) the Perceived Infectability Subscale (7 items), which assesses individuals’ beliefs about their own susceptibility to infectious diseases, and (2) the Germ Aversion Subscale (8 items), which measures emotional discomfort in situations perceived as posing a high risk of pathogen transmission. Participants respond to each item on a 7-point Likert scale (1 = strongly disagree, 7 = strongly agree), and subscale scores are calculated as the mean of the items. Example items include, “If an illness is ‘going around’, I will get it.” (perceived infectability), “It really bothers me when people sneeze without covering their mouths.” (germ aversion).

Both subscales demonstrated good internal consistency in the present study: Cronbach’s alpha was 0.88 for Perceived Infectability and 0.76 for Germ Aversion. Given that the constructs measured by the PVD are conceptualized as relatively stable traits, the scale was administered only at the first time point.

The Depression, Anxiety, and Stress Scale—21 items (DASS-21) was used to assess psychological distress, based on the Croatian adaptation of the instrument [61,62]. The DASS-21 consists of 21 self-report items grouped into three subscales that measure the negative emotional states of depression, anxiety, and stress. Respondents rated how much each statement applied to them over the past week using a 4-point Likert scale (0 = “Did not apply to me at all” to 3 = “Applied to me very much or most of the time”). Higher scores on each subscale indicate greater levels of depression, anxiety, or stress. Example items include, “I couldn’t seem to experience any positive feeling at all” (depression), “I felt close to panic” (anxiety), and “I found it hard to wind down” (stress).

The internal consistency of the subscales in the present study was high, with Cronbach’s alpha coefficients of 0.92 for depression, 0.90 for anxiety, and 0.92 for stress. The DASS-21 was administered at both time points of the study.

The Padua Inventory of obsessive–compulsive disorder symptoms [63] is a self-report measure assessing obsessive and compulsive symptoms. While the original scale comprises five subscales, only the Contamination Obsessions and Washing Compulsions Subscale was used in the present study (example item: “I sometimes have to wash or clean myself simply because I think I am contaminated”). The internal consistency of this subscale in the present study was high, with a Cronbach’s alpha of 0.89.

Additionally, participants were asked to rate how much the intensity of these symptoms had changed since the onset of the pandemic, using a scale from 1 (“not at all”) to 5 (“extremely”). This information was incorporated by computing a weighted total score. The scale was administered only at the second time point.

### 2.3. Data Analysis

All analyses were conducted using IBM SPSS Statistics (version 25; IBM Corp., Armonk, NY, USA). A series of hierarchical regression analyses was performed: specifically, two sets for each of the following dependent variables—depression, anxiety, and stress (one per time point), and one for obsessive–compulsive symptoms, as this measure was administered only at the second time point. In all cases, predictor variables were those assessed at the first time point (May 2020). Sociodemographic and socioeconomic variables (gender, age, education, income, relationship status, and parental status), along with general quality of life, were entered in the first step, followed by behavioral immune system (BIS) variables—perceived infectability and germ aversion—in the second step.

This approach was based on the rationale that SES is a well-established predictor of health outcomes. Therefore, these variables were entered first to control for their effects before examining the additional predictive value of BIS-related variables. While some authors [64] have argued against including demographic variables that are not central to the hypothesis, in this context, SES functions as a theoretically and empirically justified control. Entering BIS variables in the second block allowed us to assess their unique contribution to mental health outcomes above and beyond the influence of SES and related factors.

Categorical variables were dummy coded. For gender, 1 = men, 2 = women. Parental status was coded as 0 = no children, 1 = has children. Relationship status was coded as an ordinal variable reflecting increasing levels of presumed social support: 1 = single, 2 = in a relationship but not cohabiting, and 3 = in a cohabiting relationship (e.g., married or living with a partner). Financial status was treated as a continuous variable, with higher scores indicating better financial standing.

## 3. Results

### 3.1. Stress Levels

As can be seen from Table 2, for the first time point (April 2020), sociodemographic variables and general quality of life entered in step one accounted for 22.3% of the variation in experienced stress levels. Women, younger people, participants with lower income, singles and participants with generally lower quality of life reported significantly higher stress scores. Adding perceived vulnerability to disease and germ aversion in the second step explained an additional 2.1% of stress variance. This change was significant (*F* (2, 936) = 12.98; *p* < 0.001). Participants with higher perceived infectability and germ aversion experienced significantly higher levels of stress. All predictors except education were significant predictors in the final model, with general quality of life and gender having the highest contributions. Together they accounted for 23.7% of the variance in experienced stress levels.

Stress levels were assessed again at the end of the first pandemic year, during November and December 2020. Sociodemographic variables and general quality of life entered in step one accounted for 14.7% of the variation in experienced stress levels. With this time delay, only general quality of life remained a significant independent predictor. Adding perceived vulnerability to disease and germ aversion in the second step explained an additional 7.7% of stress variance. This change was significant (*F* (2, 171) = 12.53; *p* < 0.001). Participants with higher perceived infectability experienced significantly higher levels of stress. The general quality of life, age and perceived infectability were the only significant predictors in the final model, accounting for a total of 22.4% variance in experienced stress levels.

### 3.2. Anxiety

As can be seen from Table 3, for the first time point (April 2020), sociodemographic variables and general quality of life entered in step one accounted for 16.9% of the variation in anxiety. Women, younger people, participants with lower levels of education, singles and participants with a generally lower quality of life reported significantly higher levels of anxiety. Adding perceived vulnerability to disease and germ aversion in the second step explained an additional 3.6% of stress variance. This change was significant (*F* (2, 936) = 27.11; *p* < 0.001). Participants with higher perceived infectability and germ aversion experienced significantly higher levels of anxiety. All predictors except financial status and parental status were significant predictors in the final model, with general quality of life again having the highest contribution. Together they accounted for 20.5% of the variance in anxiety.

For the second time point (December 2020), sociodemographic variables and general quality of life entered in step one accounted for 17.1% of the variation in anxiety. With this time delay, only financial status and parental status were significant independent predictors. Adding perceived vulnerability to disease and germ aversion in the second step explained an additional 10.7% of anxiety. This change was significant (*F* (2, 171) = 12.53; *p* < 0.001). Participants with higher perceived infectability experienced significantly higher levels of anxiety. The general quality of life, relationship status, perceived infectability and germ aversion were the only independent significant predictors in the final model, accounting for a total of 27.8% variance in anxiety.

### 3.3. Depression

As can be seen from Table 4, for the first time point (May 2020), sociodemographic variables and general quality of life entered in step one accounted for 35.8% of the variation in depressive symptoms. Women, participants with a lower level of education and income, singles and participants with a generally lower quality of life reported significantly higher levels of depression. Adding perceived vulnerability to disease and germ aversion in the second step explained an additional 0.8% of stress variance. This change was significant (*F* (2, 936) = 6.02; *p* < 0.01). Participants with higher germ aversion experienced significantly higher levels of depressive symptoms. Gender, education, financial status, general quality of life and germ aversion were significant predictors in the final model, with general quality of life again having the highest contribution. Together they accounted for 36.6% of the variance in depression.

For the second time point (November/December 2020), sociodemographic variables and general quality of life entered in step one accounted for 19.6% of the variation in depression. With this time delay, the general quality of life was the only significant independent predictor. Adding perceived vulnerability to disease and germ aversion in the second step explained an additional 2.9% of the variance in depression. This change was significant (*F* (2, 171) = 3.12; *p* < 0.05). Participants with higher perceived infectability experienced higher levels of stress. The general quality of life and perceived infectability were the only independent significant predictors in the final model, accounting for a total of 22.4% variance in depression.

### 3.4. Obsessive-Compulsive Symptomatology

The Padua Inventory of obsessive–compulsive symptoms was administered during the second time point only. As can be seen in Table 5, the sociodemographic variables and general quality of life entered in the first step did not account for a significant proportion of variance in OC symptoms. However, the behavioral immune system variables entered in the second step yielded a significant change (*F* (2, 110) = 12.32; *p* < 0.001). Participants with higher germ aversion and perceived infectability at the beginning of the COVID-19 pandemic were more likely to develop OC symptoms by the end of the first pandemic year. In total, the model explained 21.8% of the variance in OC symptoms, with perceived infectability and germ aversion being the independent significant predictors.

## 4. Discussion

The findings of the present study contribute to the growing body of literature exploring the relationship between the behavioral immune system (BIS) and mental health, particularly within the context of socioeconomic status (SES). The detrimental impact of environmental and socioeconomic stressors—such as financial instability, poor housing conditions, limited access to healthcare, job insecurity, and low social support—on both physical and mental health, as well as longevity, has been well documented [39,40,41,42,50,51,52,53,54,55,65,66]. In this study, we included several such sociodemographic predictors along with a single-item measure of general life satisfaction. One-item indicators of well-being have been shown to possess strong psychometric validity and often explain more variance in general well-being than longer or more complex measures [67,68,69].

Our regression analyses support these prior findings. In the first step, higher financial and educational status were associated with better mental health outcomes (i.e., lower depression, anxiety, and stress), as was being in a romantic relationship—likely due to increased social support, which facilitates coping with environmental stressors. However, when predicting mental health outcomes over time—specifically, 6–7 months after the initial assessment—only general quality of life remained a consistent and significant predictor across both time points and all three mental health indicators. One potential explanation is adaptation and habituation: while individuals may initially respond strongly to acute stressors, sensitivity tends to decrease as they adjust to ongoing environmental conditions. Alternatively, fluctuations in psychological states and measurement error may have contributed to the observed variability. Future studies should seek to clarify these mechanisms through longitudinal and experimental designs that better disentangle causality.

Unlike the socioeconomic variables, BIS-related measures remained robust predictors of mental health across both time points. This highlights the central aim of the current study: examining the unique contribution of BIS reactivity above and beyond the effects of objective socioeconomic context.

Although the adaptive benefits of a functional BIS are well recognized, its potential downsides—especially in the domain of mental health—have received comparatively less attention. While prior studies have discussed the BIS in relation to social cognition and xenophobic attitudes [3,4,11,13,15,17,18,19,20], its role in psychological well-being has often been overlooked. During the COVID-19 pandemic, several studies demonstrated that BIS sensitivity predicted fear of infection [27,70,71], including among healthcare workers [72]. Other research confirmed that perceived vulnerability to disease predicted poorer mental health outcomes [73,74]; however, these studies failed to consider the germ aversion component of the BIS and did not frame their analyses from an evolutionary perspective.

Our findings showed that both germ aversion and perceived infectability significantly predicted levels of stress and anxiety at both time points. In the case of depression, germ aversion emerged as a significant predictor early in the pandemic, while perceived infectability became more predictive by the end of the first pandemic year. Germ aversion reflects an affective, immediate response, likely to contribute to initial emotional reactions upon learning about a highly contagious virus. Perceived infectability, being a more cognitive construct, may have grown in influence as the pandemic progressed. Importantly, both variables consistently predicted stress and anxiety over time, indicating a clear pattern of potential harm to mental health under conditions of heightened vigilance. While the amount of variance explained by these variables may appear modest, the consistency of their effects across time points and after controlling for both objective and subjective life quality indicators is notable.

The relevance of BIS measures was particularly evident in the prediction of obsessive–compulsive (OC) symptoms. In this domain, none of the sociodemographic variables included in the first regression step were significant; only germ aversion and perceived infectability made unique, statistically significant contributions. This provides further support for the notion that even highly functional adaptations carry potential psychological costs. While pathogen vigilance has historically conferred survival benefits, especially in environments rife with infectious disease, the chronic threat messaging during the COVID-19 pandemic represents a departure from typical ancestral conditions. Compared to pre-pandemic data, participants exhibited elevated levels of germ aversion, perceived infectability, pathogen disgust, and preferred interpersonal distance [34]. Certain populations may be particularly vulnerable to these effects—especially those with pre-existing mental health issues, hypersensitive BIS activation (e.g., chronically ill individuals, pregnant women, psychiatric patients), or poor quality of life.

The field of psychoneuroimmunology has uncovered a range of biological mechanisms linking affective and cognitive processes to physical health [37]. These proximal mechanisms provide useful targets for interventions aimed at reducing the negative health impacts of chronic stress. With the present study, we aimed to extend this understanding by contributing to the evolutionary (ultimate) level of analysis. Natural selection has furnished humans with a variety of elegant and seemingly low-cost behavioral adaptations, but these come with trade-offs [75,76]. The psychological costs of BIS activation may be exacerbated in situations requiring sustained or intensified engagement of these mechanisms—such as the prolonged pathogen threat posed by COVID-19. BIS hyper-reactivity can serve an adaptive function in resource-scarce environments, where heightened disease vigilance may enhance survival. However, when coupled with socioeconomic adversity, it may also contribute to mental health vulnerabilities, highlighting a complex interplay between evolutionary adaptations and environmental context.

### Limitations of the Study and Directions for Future Research

Our study was exploratory in nature. Conducted online during the COVID-19 pandemic and amid epidemiological restrictions, both the sampling process and the conditions under which participants completed the survey were beyond the researchers’ control. The sample may have been subject to bias due to self-selection and the varying willingness of certain groups to participate. This is clearly reflected in the overrepresentation of women (86% of the sample), though similar biases may have influenced other variables as well.

Additionally, both the BIS-related variables and mental health measures relied on self-report instruments, which may be affected by biases such as social desirability or participants’ emotional state at the time of data collection. While we attempted to assess socioeconomic status (SES) across multiple dimensions, SES is inherently complex and multifaceted, encompassing income, education, occupation, and neighborhood factors. Our measures may have overlooked important nuances in how SES influences BIS activity and health outcomes. Moreover, unmeasured factors such as chronic illness, personality traits, or perceived social support could influence both BIS reactivity and mental health, making it difficult to rule out potential confounders and thus limiting the specificity of our conclusions.

Finally, as our sample was drawn from a single cultural and geographical context, the findings may not be generalizable to other populations where baseline quality of life, disease prevalence, and BIS responsiveness may differ.

Despite these limitations, our results suggest that heightened BIS sensitivity may represent a risk factor for anxiety, obsessive–compulsive symptoms, and depression. Future research should investigate the neurobiological and physiological mechanisms underlying this relationship. Mental health practitioners may also benefit from examining whether BIS hyper-reactivity contributes to vulnerability to specific mental disorders and whether interventions targeting BIS-related responses (e.g., cognitive-behavioral therapy) can mitigate associated mental health risks.

In a complementary line of inquiry, cross-cultural studies could examine how the relationship between BIS and mental health varies across different cultural contexts—particularly in societies with differing levels of pathogen prevalence. Importantly, the COVID-19 pandemic provided a unique, ecologically valid opportunity to study changes in BIS reactivity in real time. These findings may inform future public health strategies: there appears to be a delicate balance between raising awareness of infectious disease threats and inadvertently exacerbating mental health issues—potentially through increased germ aversion and health anxiety.

## 5. Conclusions

In conclusion, this study demonstrated that, in addition to general socioeconomic status and overall quality of life (as contextual factors), variations in behavioral immune system (BIS) sensitivity significantly contributed to mental health outcomes during the COVID-19 pandemic. Higher levels of germ aversion and perceived infectability predicted poorer mental health outcomes both at the onset of the pandemic and at the end of its first year.

Regarding anxiety, depression, and stress, individuals with lower income, education, and social support emerged as the most vulnerable—consistent with numerous previous findings. However, even after controlling for these factors, BIS sensitivity variables remained significant predictors of mental health outcomes. In the case of obsessive–compulsive symptomatology, BIS sensitivity variables were the only significant predictors, highlighting the potential adverse side effects of these otherwise adaptive, prophylactic mechanisms.

Although perceived vulnerability to disease is typically considered a stable, trait-like characteristic, elevated scores on these measures during the COVID-19 pandemic suggest that sustained BIS activation may have contributed—at least in part—to the observed rise in mental health difficulties. These findings underscore the importance of considering both environmental and evolved psychological mechanisms in understanding the mental health impacts of large-scale public health crises.

## Figures and Tables

**Table 1:** Demographic characteristics of the sample.

	First Time Point (*n* = 990)	Second Time Point (*n* = 182, a Subsample of the Original Sample)
Gender		
men	132 (13.3%)	25 (13.7%)
women	855 (86.4%)	156 (85.7%)
other	3 (0.3%)	1 (0.5%)
Age	*M* = 36.45 (*SD* = 14.24; range: 18–80)	*M* = 34.91 (*SD* = 13.99; range: 19–77)
Education		
primary school	13 (1.3%)	2 (1.1%)
high school	261 (26.4%)	48 (26.4%)
undergraduate	240 (24.2%)	36 (19.8%)
graduate	341 (34.4%)	73 (40.1%)
PhD	135 (13.6%)	23 (12.6%)
Relationship status		
single	396 (40.0%)	71 (39.0%)
in a relationship, but not cohabitating	185 (18.7%)	39 (21.4%)
in a cohabitating relationship (marriage/partnership)	409 (41.3%)	72 (39.6%)

**Table 2:** Summary of hierarchical regression analyses for variables predicting stress levels.

Dependent Variable: Stress										
	First Time Point (May 2020)					Second Time Point (November/December 2020) Prediction from the First Time Point				
	β	* t *	* p *	*R* ^2 ^	Δ*R*^2^	β	* t *	* P *	*R* ^2 ^	Δ*R*^2^
Step 1										
Gender	0.181	6.23	<0.001	0.223 ***	0.223 ***	0.053	0.72	0.470	0.147 ***	0.147 ***
Age	−0.129	−3.13	0.002			−0.194	−1.86	0.065		
Education	−0.053	−1.58	0.116			0.062	0.74	0.463		
Financial status	−0.095	−3.08	0.002			−0.066	−0.84	0.402		
Relationship status	−0.124	−3.62	<0.001			−0.107	−1.45	0.148		
Parental status	0.075	1.79	0.074			−0.012	−0.12	0.907		
General quality of life	−0.386	−12.74	<0.001			−0.312	−4.13	<0.001		
Step 2										
Gender	0.165	5.71	<0.001	0.237 ***	0.021 ***	0.022	0.31	0.760	0.224 ***	0.077 ***
Age	−0.147	−3.56	<0.001			−0.219	−2.79	0.031		
Education	−0.046	−1.38	0.169			0.070	0.85	0.397		
Financial status	−0.093	−3.04	0.002			−0.053	−0.70	0.485		
Relationship status	−0.117	−3.44	0.001			−0.093	−1.30	0.195		
Parental status	0.083	1.99	0.046			−0.034	−0.35	0.728		
General quality of life	−0.367	−12.11	<0.001			−0.274	−3.75	<0.001		
Perceived infectability	0.082	2.74	0.006			0.225	3.20	0.002		
Germ aversion	0.104	3.47	<0.001			0.132	1.85	0.066		

*Note*: *** *p* < 0.001. Categorical variables were dummy coded. For gender, 1 = men; 2 = women. For parental status, 0 = no children, 1 = has children. Relationship status was coded on an ordinal scale: 1 = single; 2 = in a relationship but not cohabiting; 3 = in a cohabiting relationship (e.g., married or living with a partner). Financial status was treated as a continuous variable, with higher values indicating better financial standing.

**Table 3:** Summary of hierarchical regression analyses for variables predicting anxiety.

Dependent Variable: Anxiety										
	First Time Point (May 2020)					Second Time Point (November/December 2020) Prediction from the First Time Point				
	β	* t *	* p *	*R* ^2 ^	Δ*R*^2^	β	* t *	* p *	*R* ^2 ^	Δ*R*^2^
Step 1						0.118	1.62	0.106		
Gender	0.145	4.82	<0.001	0.169 ***	0.169 ***	−0.126	−1.23	0.219	0.171 ***	0.171 ***
Age	−0.097	−2.27	0.024			−0.041	−0.49	0.624		
Education	−0.095	−2.69	0.007			0.013	0.17	0.869		
Financial status	−0.055	−1.70	0.090			−0.172	−2.36	0.019		
Relationship status	−0.121	−3.41	<0.001			−0.035	−0.35	0.726		
Parental status	0.047	1.07	0.283			−0.264	−3.55	0.001		
General quality of life	−0.339	−10.77	<0.001			0.118	1.62	0.106		
Step 2										
Gender	0.124	4.20	<0.001	0.205 ***	0.036 ***	0.082	1.20	0.231	0.278 ***	0.107 ***
Age	−0.121	−2.84	0.005			−0.170	−1.75	0.082		
Education	−0.085	−2.45	0.015			−0.022	−0.28	0.780		
Financial status	−0.052	−1.64	0.101			−0.009	−0.12	0.902		
Relationship status	−0.111	−3.20	<0.001			−0.148	−2.16	0.032		
Parental status	0.056	1.30	0.195			−0.058	−0.62	0.535		
General quality of life	−0.314	−10.08	<0.001			−0.222	−3.14	0.002		
Perceived infectability	0.099	3.23	<0.001			0.204	3.01	0.003		
Germ aversion	0.143	4.64	<0.001			0.228	3.31	<0.001		

*Note*: *** *p* < 0.001. Categorical variables were dummy coded. For gender, 1 = men; 2 = women. For parental status, 0 = no children, 1 = has children. Relationship status was coded on an ordinal scale: 1 = single; 2 = in a relationship but not cohabiting; 3 = in a cohabiting relationship (e.g., married or living with a partner). Financial status was treated as a continuous variable, with higher values indicating better financial standing.

**Table 4:** Summary of hierarchical regression analyses for variables predicting depression.

Dependent Variable: Depression										
	First Time Point (May 2020)					Second Time Point (November/December 2020) Prediction from the First Time Point				
	β	* t *	* p *	*R* ^2 ^	Δ*R*^2^	β	* t *	* p *	*R* ^2 ^	Δ*R*^2^
Step 1										
Gender	0.093	3.53	<0.001	0.358 ***	0.358 ***	0.021	0.29	0.772	0.196 ***	0.196 ***
Age	−0.060	−1.61	0.108			−0.146	−1.44	0.151		
Education	−0.078	−2.53	0.011			0.022	0.26	0.793		
Financial status	−0.070	−2.50	0.013			−0.092	−1.28	0.202		
Relationship status	−0.064	−2.05	0.041			−0.016	−0.21	0.831		
Parental status	0.025	0.65	0.518			0.026	0.27	0.792		
General quality of life	−0.561	−20.35	<0.001			−0.404	−5.51	<0.001		
Step 2										
Gender	0.083	3.14	0.002	0.366 ***	0.008 **	0.002	0.03	0.974	0.224 ***	0.029 *
Age	−0.073	−1.92	0.055			−0.149	−1.48	0.141		
Education	−0.073	−2.37	0.018			0.018	0.212	0.828		
Financial status	−0.069	−2.46	0.014			−0.088	−1.24	0.216		
Relationship status	−0.059	−1.92	0.055			−0.014	−0.18	0.855		
Parental status	0.029	0.77	0.441			0.012	0.12	0.902		
General quality of life	−0.550	−19.79	<0.001			−0.382	−5.23	<0.001		
Perceived infectability	0.045	1.66	0.097			0.167	2.38	0.018		
Germ aversion	0.069	2.53	0.012			0.019	0.27	0.785		

*Note*: *** p < 0.001. ** *p* < 0.01. * *p* < 0.05. Categorical variables were dummy coded. For gender, 1 = men; 2 = women. For parental status, 0 = no children, 1 = has children. Relationship status was coded on an ordinal scale: 1 = single; 2 = in a relationship but not cohabiting; 3 = in a cohabiting relationship (e.g., married or living with a partner). Financial status was treated as a continuous variable, with higher values indicating better financial standing.

**Table 5:** Summary of hierarchical regression analysis for OC symptomatology.

Dependent Variable: Obsessive–Compulsive Symptoms (Padua Inventory)					
	Second Time Point (November/December 2020) Prediction from the First Time Point				
Predictors	β	* t *	* p *	*R* ^2^	Δ*R*^2^
Step 1					
Gender	−0.046	−0.477	0.634	0.043	0.043
Age	−0.119	−1.88	0.213		
Education	0.063	0.24	0.468		
Financial status	−0.148	−1.51	0.808		
Relationship status	−0.146	−1.52	0.135		
Parental status	0.058	0.09	0.471		
General quality of life	0.036	−0.03	0.787		
					
Step 2					
Gender	−0.013	−0.16	0.634	0.218 ***	0.175 ***
Age	−0.182	−1.61	0.213		
Education	0.077	0.84	0.404		
Financial status	−0.099	−1.13	0.260		
Relationship status	−0.067	−0.83	0.408		
Parental status	0.019	0.17	0.864		
General quality of life	0.066	0.74	0.460		
Perceived infectability	0.189	2.17	0.032		
Germ aversion	0.355	7.10	<0.001		

*Note*: *** *p* < 0.001. Categorical variables were dummy coded. For gender, 1 = men; 2 = women. For parental status, 0 = no children, 1 = has children. Relationship status was coded on an ordinal scale: 1 = single; 2 = in a relationship but not cohabiting; 3 = in a cohabiting relationship (e.g., married or living with a partner). Financial status was treated as a continuous variable, with higher values indicating better financial standing.

## Data Availability

The data presented in this study are available on request from the corresponding author.
